# Supplementary material for: Quorum sensing N-Acyl homoserine lactones are a new class of anti-schistosomal
Source: PLoS Negl Trop Dis. 2020 Oct 19;14(10):e0008630. doi: 10.1371/journal.pntd.0008630 (PMC7595621; doi:10.1371/journal.pntd.0008630)

**S1 Protocol**

Scheme 1: Method A: NEt_3_, CHCl_3_, 0^o^C; Method B: K_2_CO_3_, CH_2_Cl_2_/H_2_O, 0^o^C. R = H. Me, X = O, S.* Chiral centres

**Materials and Methods for Chemical synthesis of compounds 1 – 9**

All reactions were monitored by thin layer chromatography and visualised by either I_2_, UV light or phosphomolybdic acid. Flash chromatography was carried out on Fluorochem Silicagel 60Å (40-63 micron) with eluting solvent as indicated. ^1^H and ^13^C NMR spectroscopy was carried out on a Bruker Ultrashielded Plus 400 MHz spectrometer in CDCl_3_ unless otherwise stated and are reported in ppm referenced to the solvent internal standard at 7.62 and 77.160 ppm respectively. Infrared spectroscopy was carried out on a Bruker Alpha ATR using solid state compound. Mass spectrum were recorded on either a Finnigan MAT 900 XLT or a Finnigan MAT 95 XP at the EPSRC National Mass Spectrometry Service Centre in Swansea. Optical rotations were determined on an ADP440 Polarimeter.

**Method A** (Used for compounds **1, 2, 7** and **9**)

The acyl halide (1.00-2.65 equiv.) was added in a dropwise manner over 5 min to a stirred and cooled (0°C) solution of the aminolactone salt (1 equiv.) and triethylamine (2 equiv.) dissolved in chloroform. After 2 h the reaction was warmed to rt and stirred for 16 h. After evaporation, the mixture was triturated with EtOAc (3 x 10 mL), filtered, evaporated and purified via column chromatography.

**Method B** (Used for compounds **3, 4, 5, 6** and **8**)

The acyl halide (1.00-2.65 equiv.) was added in a dropwise manner over 5 min to a stirred and cooled (0°C) solution of the aminolactone salt (1 equiv.) and potassium carbonate (3. equiv.) dissolved in a mixture of chloroform and water. After 2 h the reaction was warmed to rt and stirred for 16 h whereupon the organic layer was separated, and the aqueous phase extracted with chloroform (20 mL). The combined organic layers were washed with tartaric acid solution (5% aq., 2 x 5 mL) and water (2 x 5 mL), dried over MgSO_4_, evaporated, and purified by column chromatography.

**Compound 1: 2-bromo-N-(2-oxotetrahydrofuran-3-yl)acetamide 1^1^**

Bromoacetyl bromide (1.47 g, 7.27 mmol, 0.63 mL), α-amino-γ-butyrolactone hydrobromide (500 mg, 2.75 mmol), triethylamine (556 mg, 5.49 mmol), in chloroform (20 mL). Column chromatography (50% EtOAc in chloroform) gave **1** (612 mg, 2.76 mmol) in 75% yield as a white solid. **δ_H_** 6.95 (1H, br s, NH), 4.56 (1H, ddd, *J* 6.1, 8.6, 11.5 Hz, CH), 4.52 (1H, br t, *J* 8.9 Hz, CH), 4.33 (1H, ddd, *J* 5.9, 9.5, 11.1 Hz), 3.96 (1H, d, *J* 14.0 Hz, CH), 3.92 (1H, d, *J* 14.0 Hz, CH), 2.84-2.92 (1H, m, CH), 2.18-2.29 (1H, m, 1H) ;** δ_C_** 174.6, 166.4, 66.2, 50.0, 30.1, 28.4; ***v*_max_** 3250, 3062, 1760, 1659, 1548, 1180; MS(CI) 222.0 (100%, [C_6_H_8_^79^BrNO_3_+H]^+^) 224.0 (98%, [C_6_H_8_^81^BrNO_3_+H]^+^), 244.0 (85%, [C_6_H_8_^79^BrNO_3_+Na]^+^), 246.0 (85%, [C_6_H_8_^79^BrNO_3_+Na]^+^); HRMS(ES) found 221.9762, C_6_H_9_^79^BrNO_3_^+^ ([M+H]^+^) requires 221.9760; Microanalysis: found C 32.6, H 3.8, N 6.4, Br 36.0; C_6_H_8_BrNO_3_ requires C 32.4, H 3.6, N 6.3, Br 36.0.


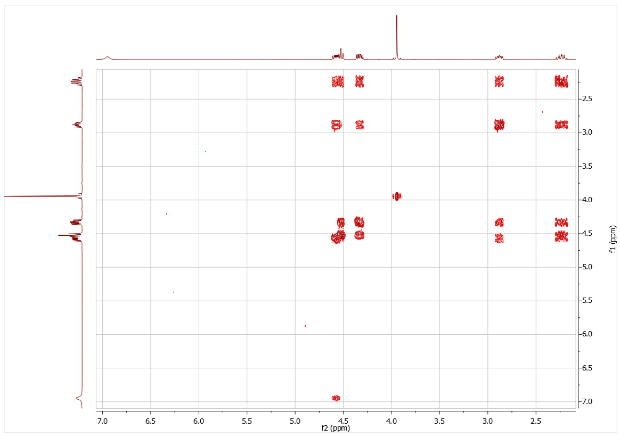

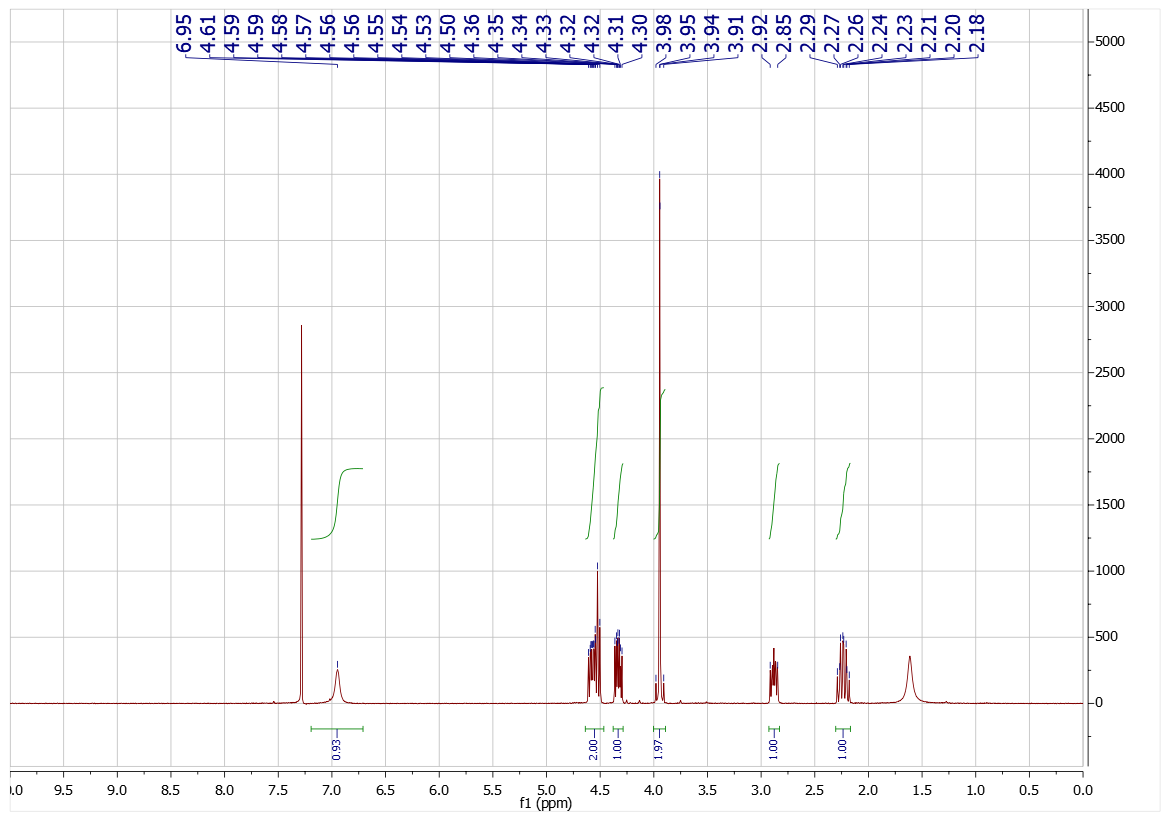

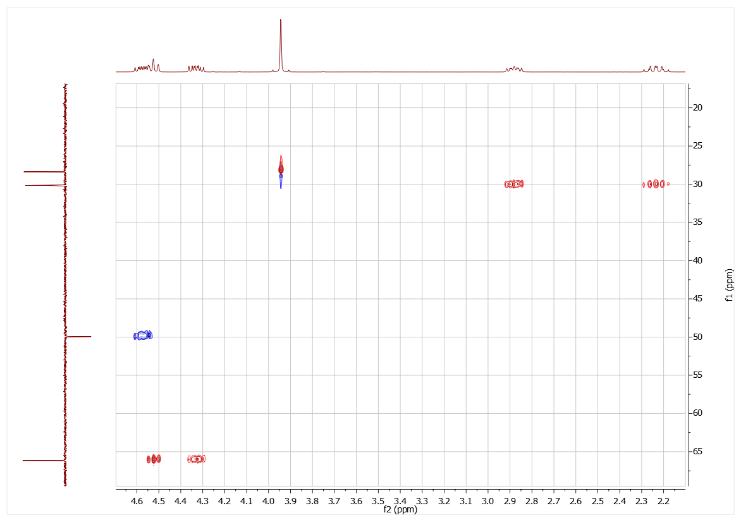

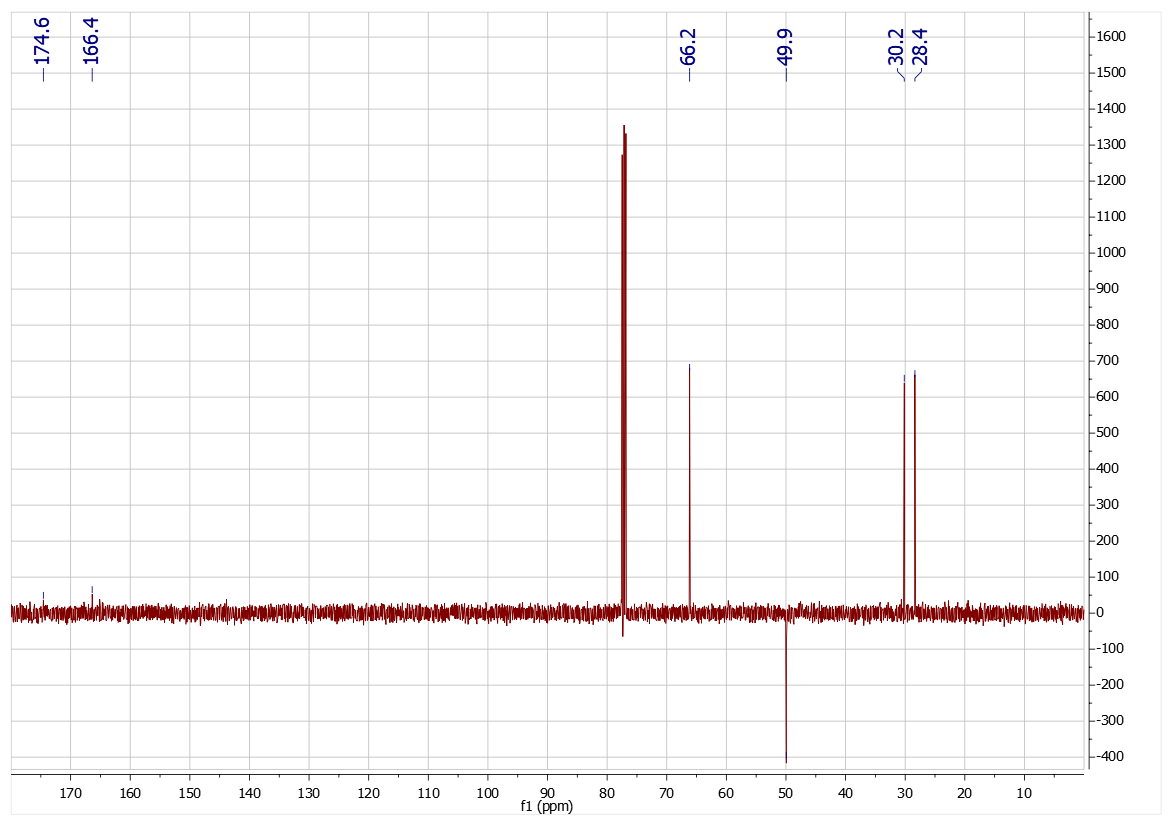

**Compound 2: 2-bromo-N-(2-oxotetrahydrofuran-3-yl)propanamide 2**

Bromopropionoyl bromide (593 mg, 2.75 mmol, 0.29 mL), α-amino-γ-butyrolactone hydrobromide (500 mg, 2.75 mmol) and triethylamine (556 mg, 5.49 mmol) in chloroform (20 mL). Column chromatography (50% EtOAc in chloroform) gave **2** (215 mg, 0.91 mmol) in 33% yield as a white solid (1:1 mixture of diasteroisomers). **δ_H_** 6.82/6.77 (1H, 2 br s, NH), 4.41-4.58 (3H, m, 3 x CH), 4.27-4.34 (1H, m, CH), 2.83-2.90 (1H, m, CH), 2.14-2.26 (1H, m, CH), 1.89/1.91 (3H, 2 x d, *J* 6.7 Hz, 2 x CH_3_); **δ_C_** 66.2, 50.0/49.9, 43.9/43.7, 30.2/30.2, 22.9/22.8 (2 x C not observed); *v*_max_ 3284, 3083, 2946, 1775, 1656, 1551, 1165; MS(CI) 236.0 (80%, [C_7_H_10_^79^BrNO_3_+H]^+^) 238.0 (80%, [C_7_H_10_^81^BrNO_3_+H]^+^), 258.0 (100%, [C_7_H_10_^79^BrNO_3_+Na]^+^), 260.0 (98%, [C_7_H_10_^81^BrNO_3_+Na]^+^); HRMS(ES) found 235.9919, C_7_H_11_^79^BrNO_3_^+^ ([M+H]^+^) requires 235.9917.


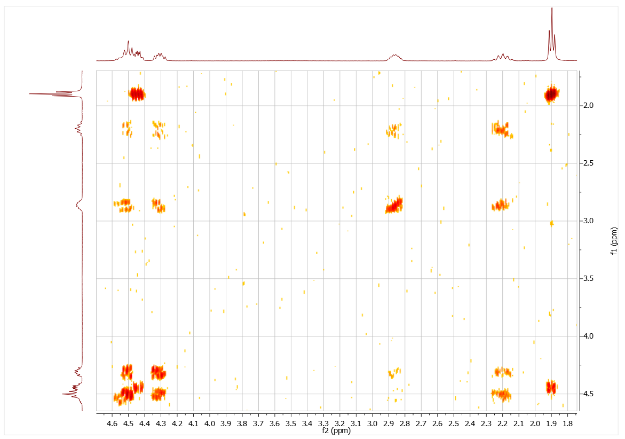

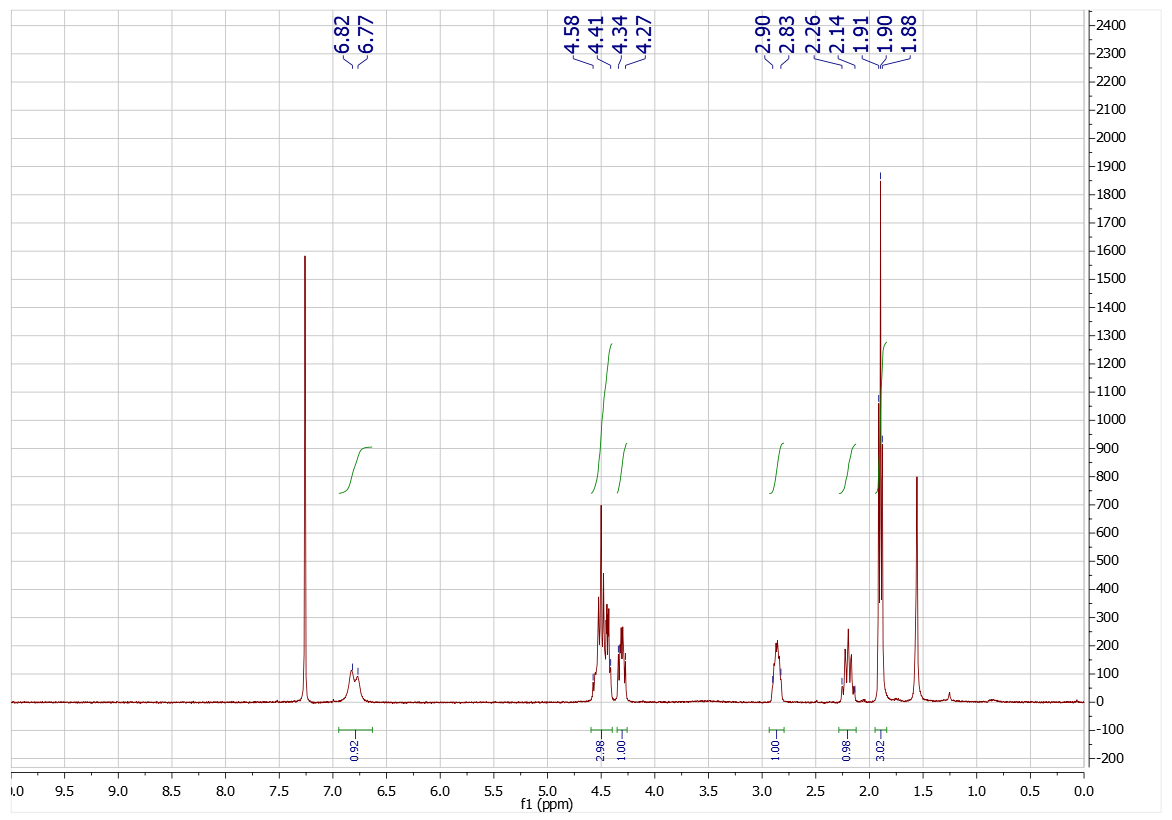

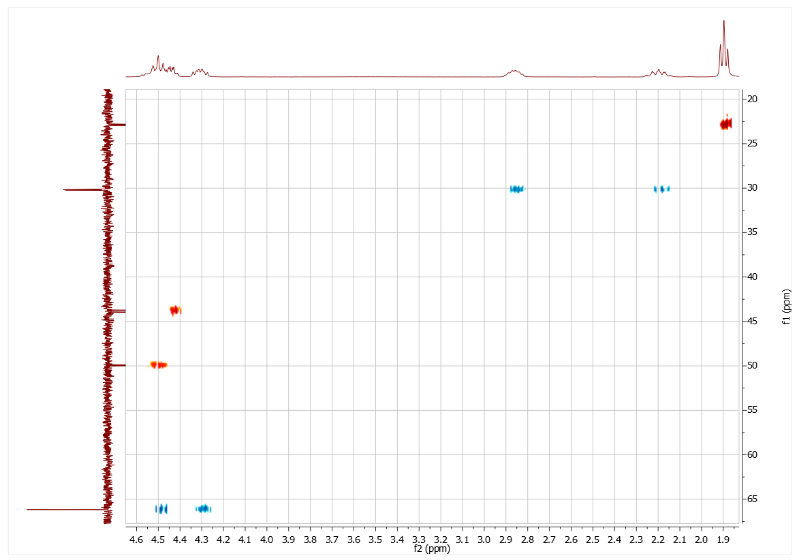

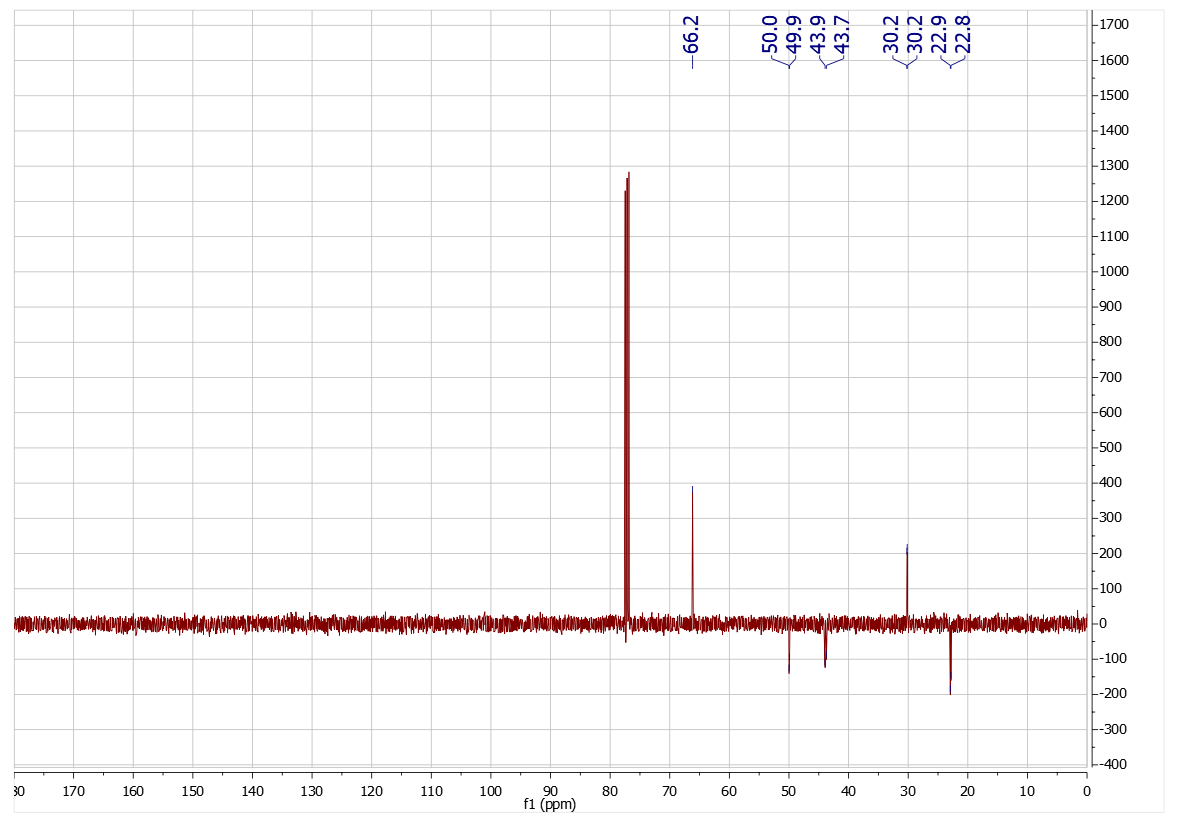

**Compound 3: 2-bromo-*N*-(2-oxotetrahydrothiophen-3-yl)acetamide 3**

Bromoacetyl bromide (1.31 g, 6.51 mmol, 0.57 mL), homocysteine thiolactone hydrochloride (500 mg, 3.25 mmol), potassium carbonate (1.35 g, 9.76 mmol), water (20 mL), chloroform (20 mL). Column chromatography (50% EA in CF) gave **3** (307 mg, 1.29 mmol) in 40% yield as a white solid. **δ_H_** 6.92 (1H, s, NH), 4.51 (1H, ddd, *J* 6.6, 6.6, 12.4 Hz, CH), 3.93 (1H, d, *J* 13.7 Hz, CH), 3.89 (1H, d, *J* 13.7 Hz, CH), 3.38 (1H, ddd, *J* 5.1, 11.6, 12.0 Hz, CH), 3.23 (1H, ddd, *J* 0.8, 7.0, 12.2 Hz, CH), 2.88-2.94 (1H, m, CH), 2.00 (dddd, *J* 7.0, 12.0, 12.2, 12.4 Hz, CH); **δ_C_** 204.6, 166.3, 59.9, 31.5, 28.6, 27.7; ***v*_max_** 3262, 1697, 1658, 1537, 1453; MS(CI) 238.0 (95%, [C_6_H_8_^79^BrNO_2_S+H]^+^) 240.0 (100%, [C_6_H_8_^81^BrNO_2_S+H]^+^), 260.0 (80%, [C_6_H_8_^79^BrNO_2_S+Na]^+^), 262.0 (85%, [C_6_H_8_^81^BrNO_2_S+Na]^+^); HRMS(ES) found 237.9534, C_6_H_9_^79^BrNO_2_S^+^ ([M+H]^+^) requires 237.9532.


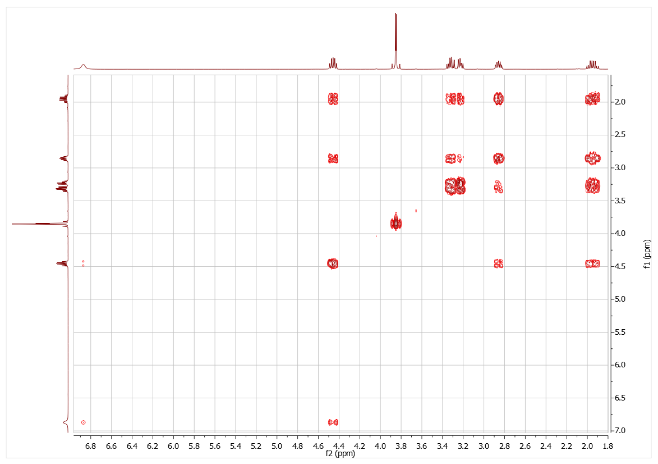

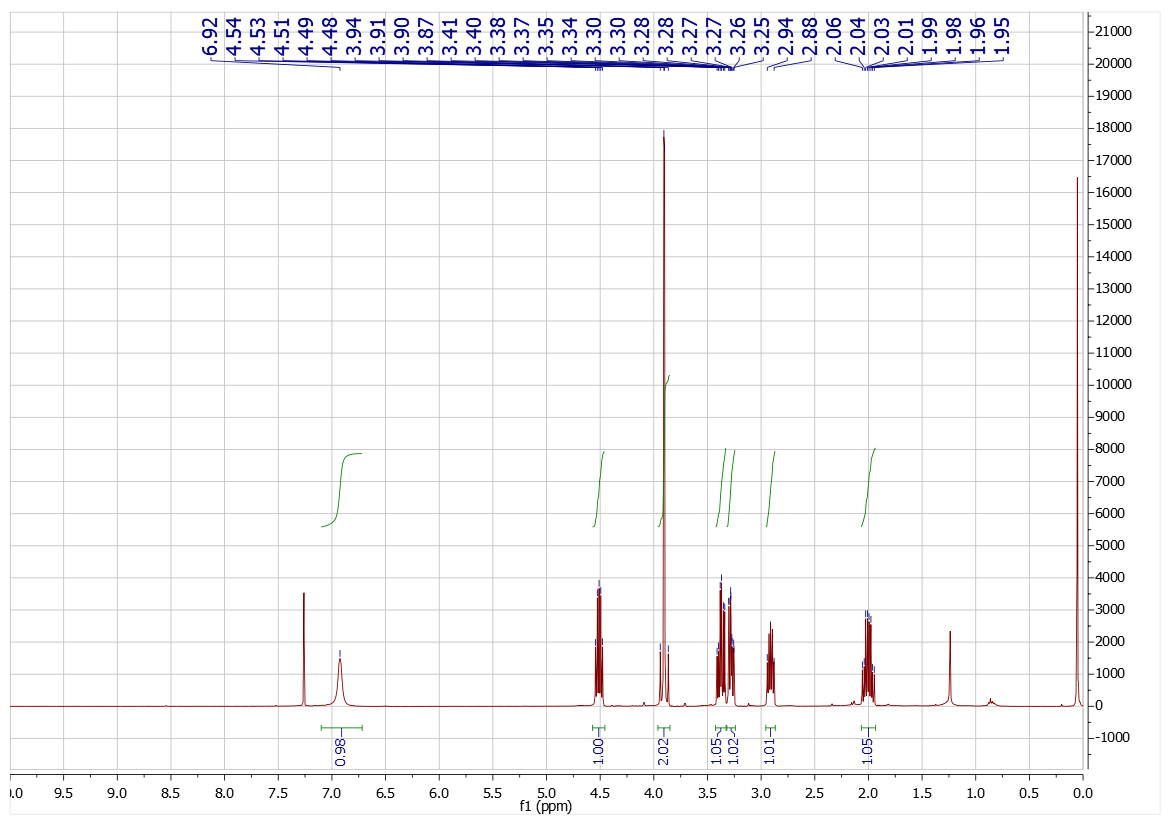

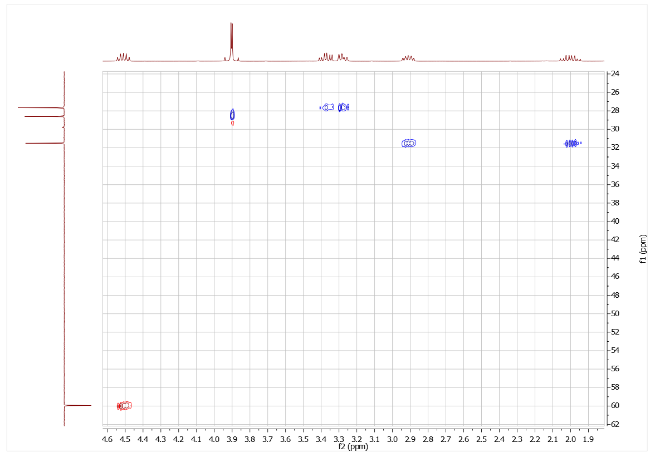

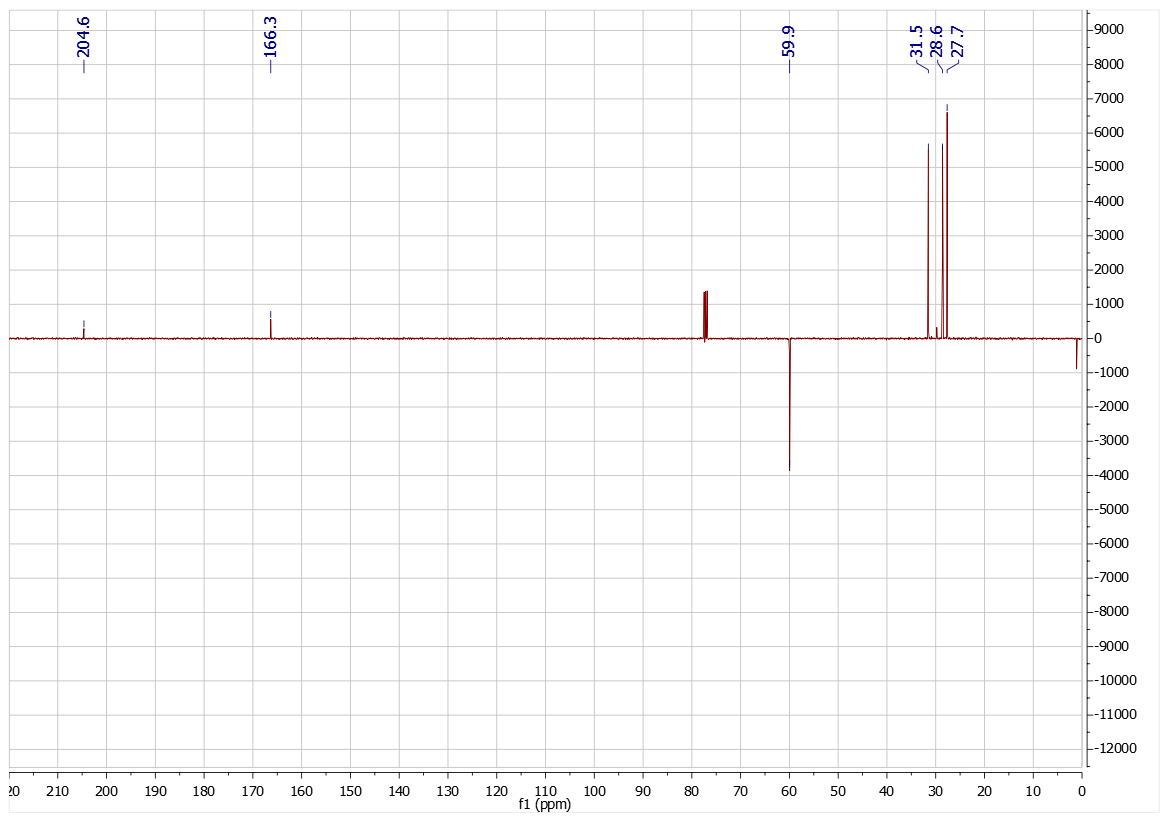

**Compound 4: 2-bromo-N-(2-oxotetrahydrothiophen-3-yl)propanamide 4**

Bromopropionyl bromide (1.41 g, 6.51 mmol, 0.68 mL), homocysteine thiolactone hydrochloride (500 mg, 3.25 mmol), potassium carbonate (1.35 g, 9.76 mmol), water (20 mL), chloroform (20 mL). Column chromatography (50% EtOAc in chloroform) gave **4** (598 mg, 2.37 mmol) in 73% yield as a white solid. **δ_H_** 6.82/6.76 (1H, 2 x br s, 2 x NH), 4.40-4.53 (1H, m, CH), 3.34-3.41 (1H, m, CH), 3.25-3.30 (1H, m, CH), 2.90-2.96 (1H, m, CH), 1.93-2.04 (1H, m, CH), 1.86/1.89 (3H, d/d, *J* 7.0/7.1 Hz, 2 x CH_3_); **δ_C_** 204.9/204.7, 170.1/170.1, 60.0/60.0, 44.2/43.9, 31.6/31.5, 27.7/27.7, 23.0/22.8; ***v*_max_** 3256, 3080, 2970, 1686, 1644, 1553; MS(CI) 252.0 (95%, [C_7_H_10_^79^BrNO_2_S+H]^+^) 254.0 (100%, [C_7_H_10_^81^BrNO_2_S+H]^+^), 269.0 (65%, [C_7_H_10_^79^BrNO_2_S+Na]^+^), 271.0 (65%, [C_7_H_10_^81^BrNO_2_S+Na]^+^); HRMS(ES) found 251.9688, C_7_H_11_^79^BrNO_2_S^+^ ([M+H]^+^) requires 251.9688.


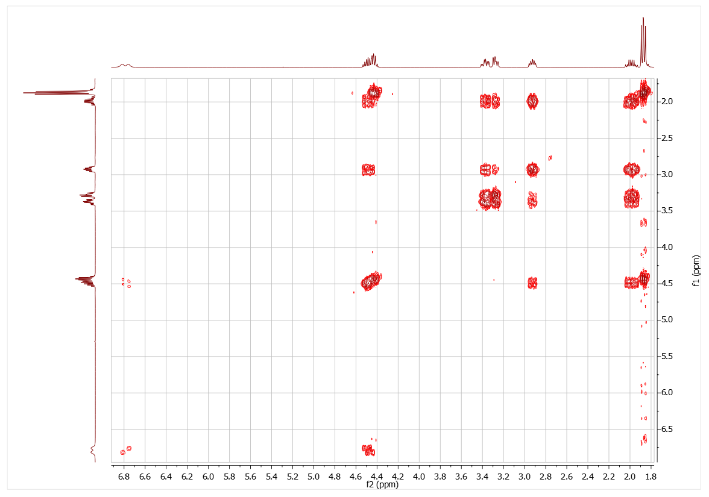

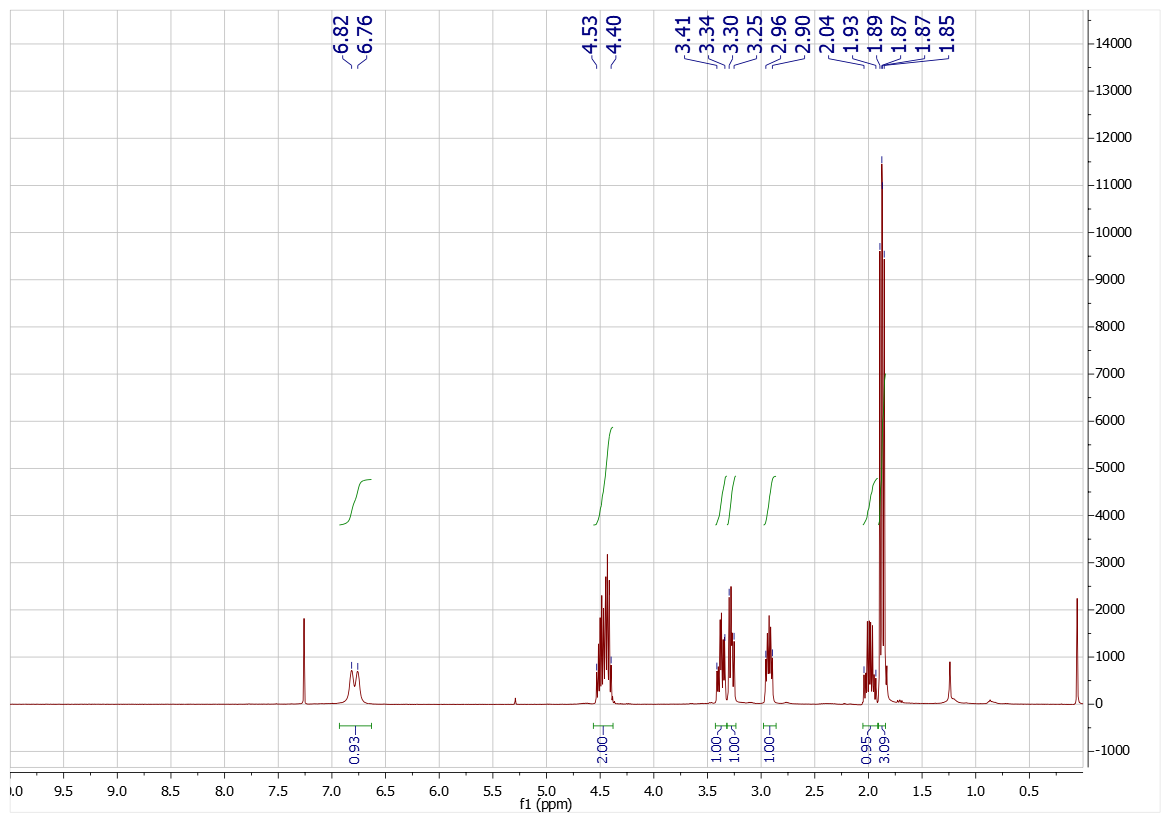


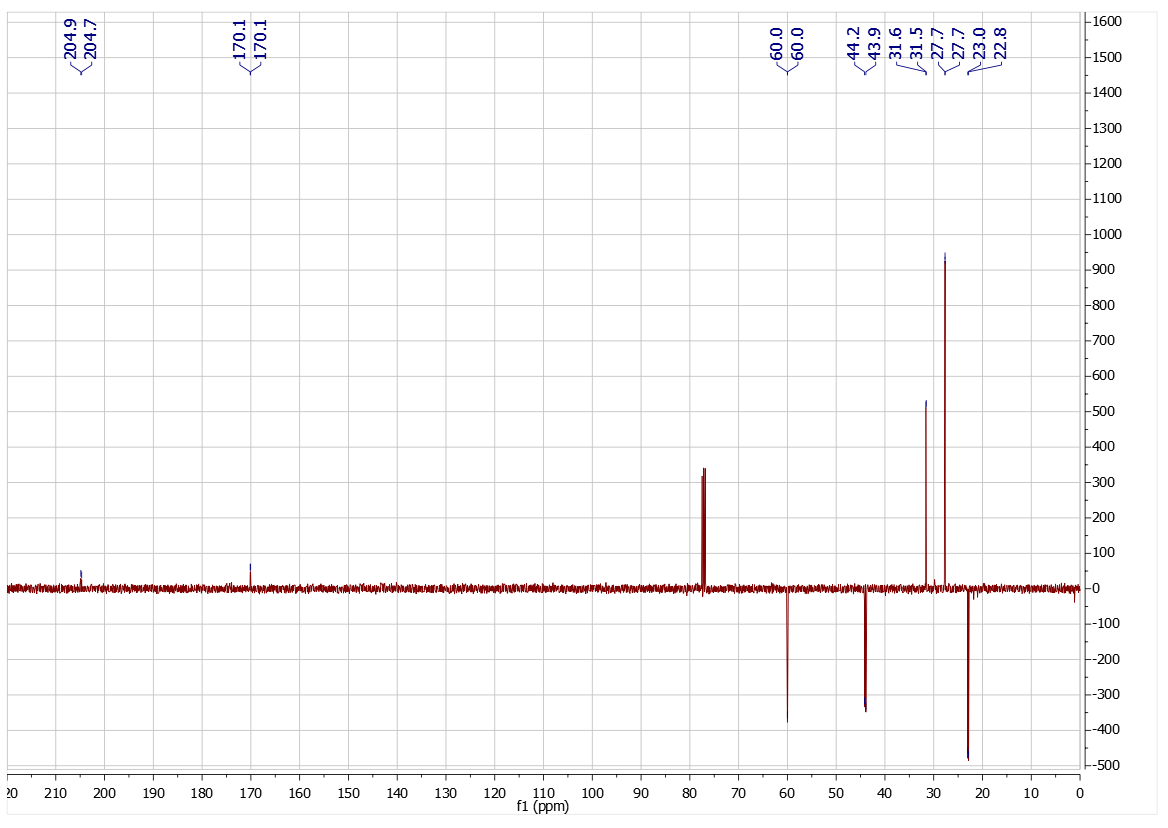


**Compound 5: (*R*)-2-bromo-N-(2-oxotetrahydrofuran-3-yl)acetamide 5**

Bromoacetyl bromide (1.47 g, 7.27 mmol, 0.63 mL), (*R*)-(+)-α-Amino-γ-butyrolactone hydrochloride (500 mg, 3.63 mmol), potassium carbonate (1.51 g, 10.90 mmol), water (20 mL), chloroform (20 mL). column chromatography (50% EA in CF) gave **5** (204 mg, 0.92 mmol) in 25% yield as a white solid. [****]_D_^19^ -22 (c = 0.1 CHCl_3_), **δ_H_** 6.91 (1H, br s, NH), 4.55 (1H, ddd, *J* 6.1, 8.6, 11.5 Hz, CH), 4.52 (1H, br t, *J* 8.9 Hz, CH), 4.34 (1H, ddd, *J* 5.9, 9.5, 11.1 Hz), 3.96 (1H, d, *J* 13.7 Hz, CH), 3.92 (1H, d, *J* 13.7 Hz, CH), 2.82-2.90 (1H, m, CH), 2.15-2.26 (1H, m, 1H);** δ_C_** 174.6, 166.4, 66.2, 50.0, 30.2, 28.4; ***v*_max_** 3253, 3064, 1762, 1657, 1551, 1179; MS(CI) 222.0 (98%, [C_6_H_8_^79^BrNO_3_+H]^+^) 224.0 (97%, [C_6_H_8_^81^BrNO_3_+H]^+^), 244.0 (98%, [C_6_H_8_^79^BrNO_3_+Na]^+^), 246.0 (98%, [C_6_H_8_^79^BrNO_3_+Na]^+^); HRMS(ES) found 221.9762, C_6_H_9_^79^BrNO_3_^+^ ([M+H]^+^) requires 221.9760.


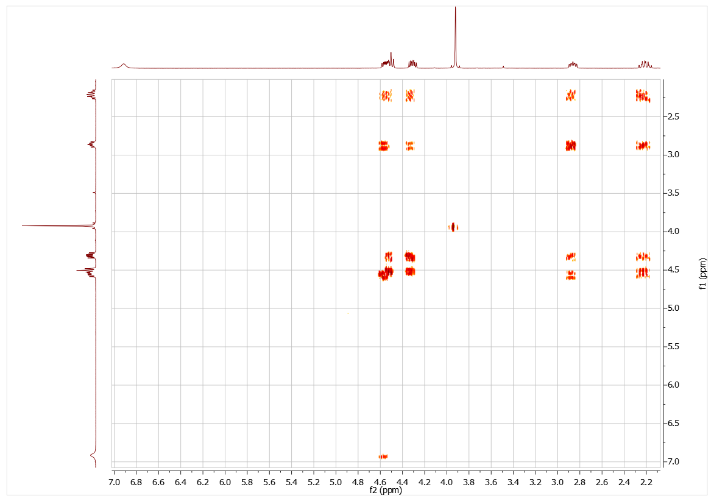

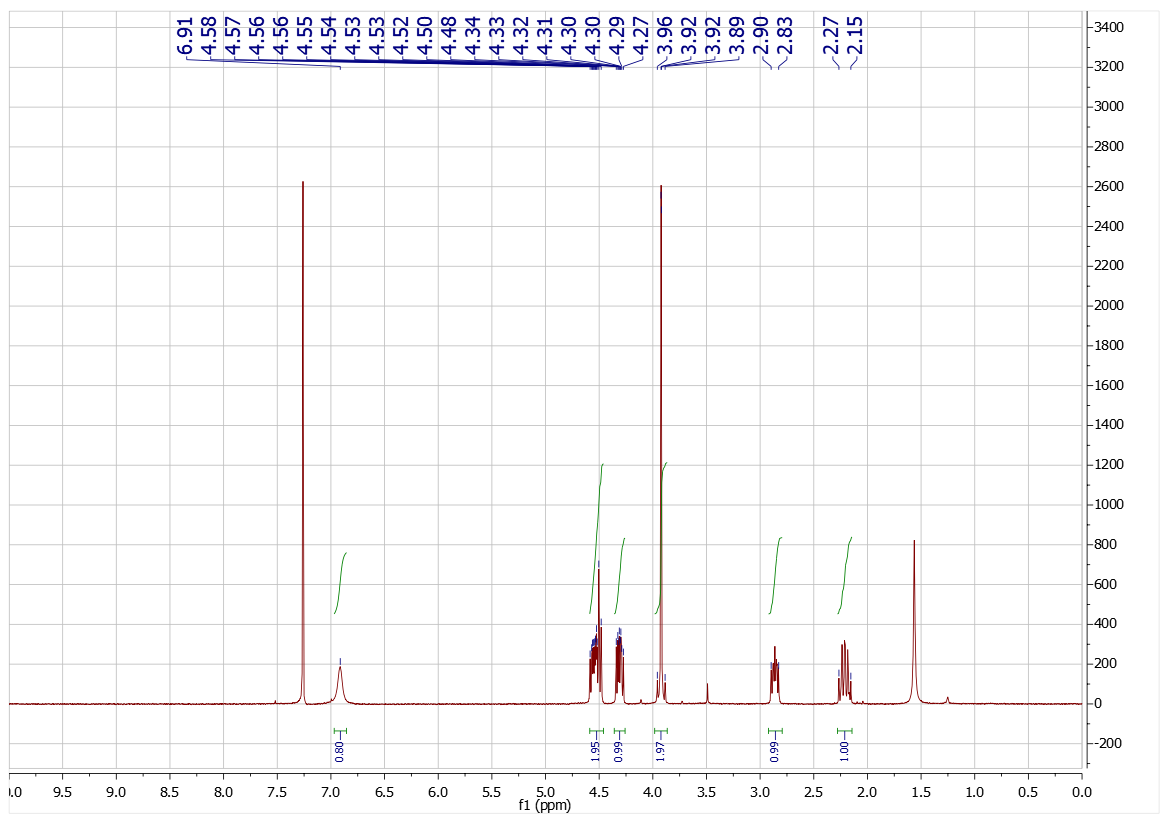

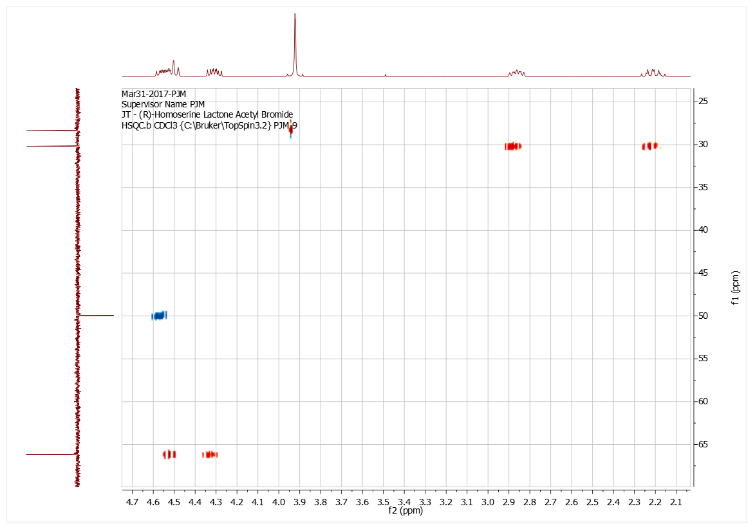

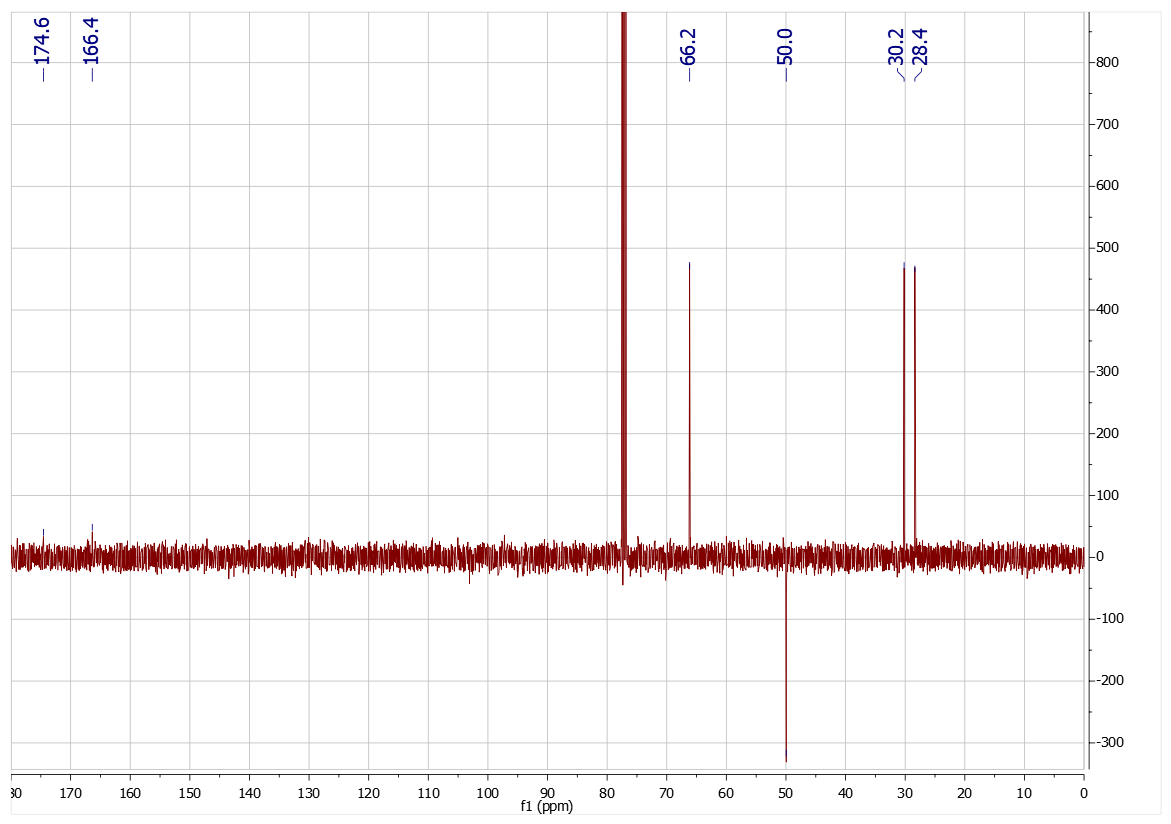

**Compound 6: (*S*)-2-bromo-N-(2-oxotetrahydrofuran-3-yl)acetamide 6^13^**

Bromoacetyl bromide (1.47 g, 7.27 mmol, 0.63 mL), (*S*)-(-)-α-Amino-γ-butyrolactone hydrochloride (500 mg, 3.63 mmol), potassium carbonate (1.35 g, 9.76 mmol), water (20 mL), chloroform (20 mL). Column chromatography (50% EA in CF) gave **6** (116 mg, 0.52 mmol) in 14% yield as a white solid. [****]_D_^19^ 21 (c = 0.1 CHCl_3_), Lit.^13^ [****]_D_^22^ 20.5 (c = 0.0074 CHCl_3_); **δ_H_** 6.99 (1H, s, NH), 4.55 (1H, ddd, *J* 6.1, 8.6, 11.5 Hz, CH), 4.52 (1H, br t, *J* 8.9 Hz, CH), 4.34 (1H, ddd, *J* 5.9, 9.5, 11.1 Hz), 3.96 (1H, d, *J* 13.7 Hz, CH), 3.92 (1H, d, *J* 13.7 Hz, CH), 2.81-2.87 (1H, m, CH), 2.17-2.28 (1H, m, 1H);** δ_C_** 174.7, 166.4, 66.2, 49.9, 30.1, 28.4; ***v*_max_** 3254, 3065, 1762, 1656, 1551, 1177; MS(CI) 222.0 (100%, [C_6_H_8_^79^BrNO_3_+H]^+^) 224.0 (98%, [C_6_H_8_^81^BrNO_3_+H]^+^), 244.0 (85%, [C_6_H_8_^79^BrNO_3_+Na]^+^), 246.0 (85%, [C_6_H_8_^79^BrNO_3_+Na]^+^); HRMS(ES) found 221.9761, C_6_H_9_^79^BrNO_3_^+^ ([M+H]^+^) requires 221.9760.


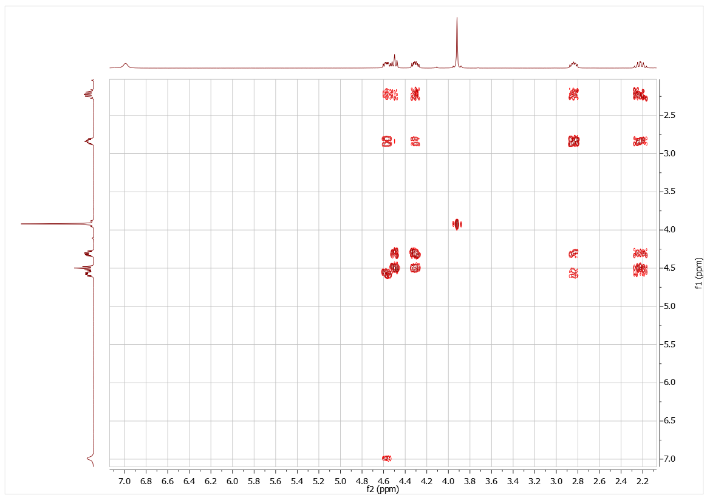

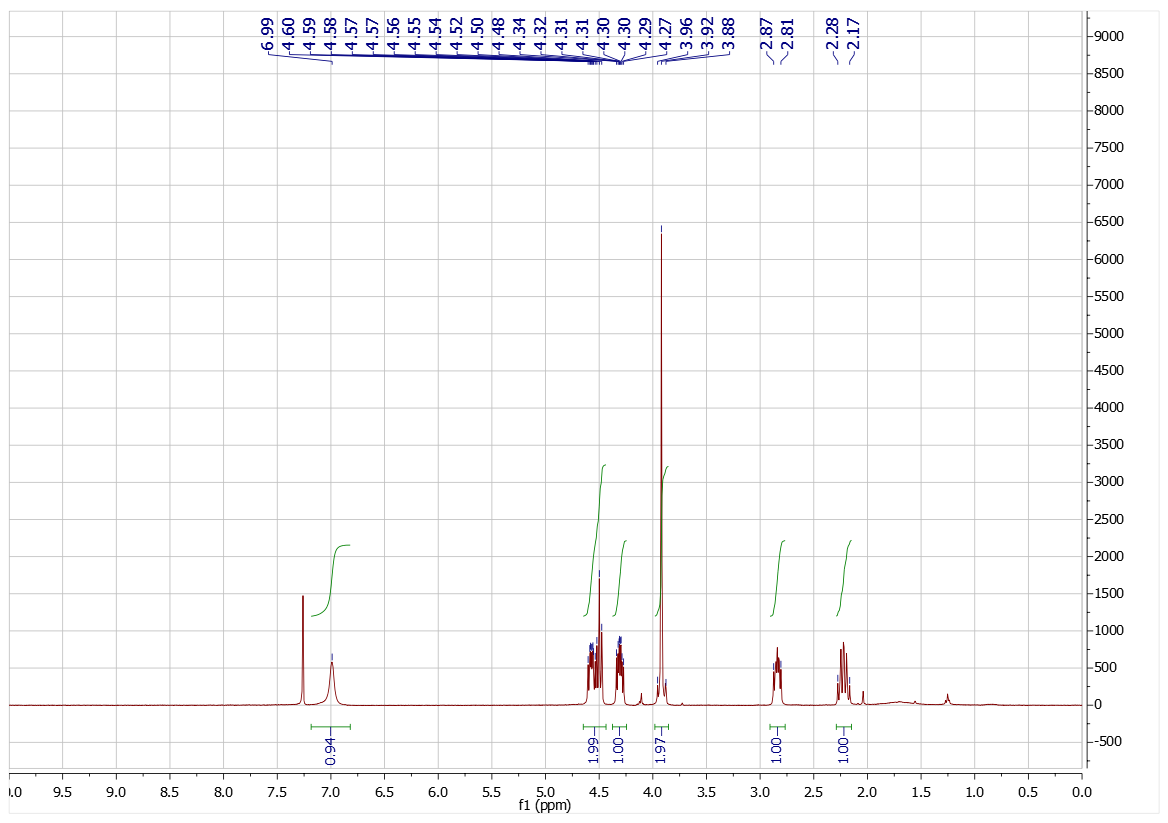

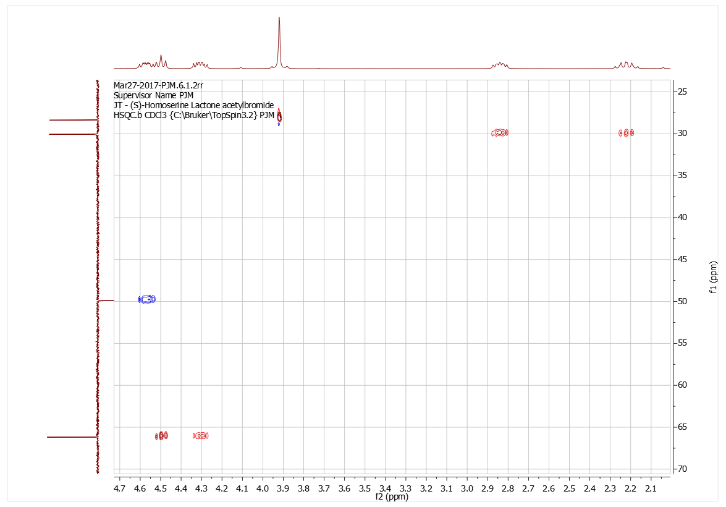

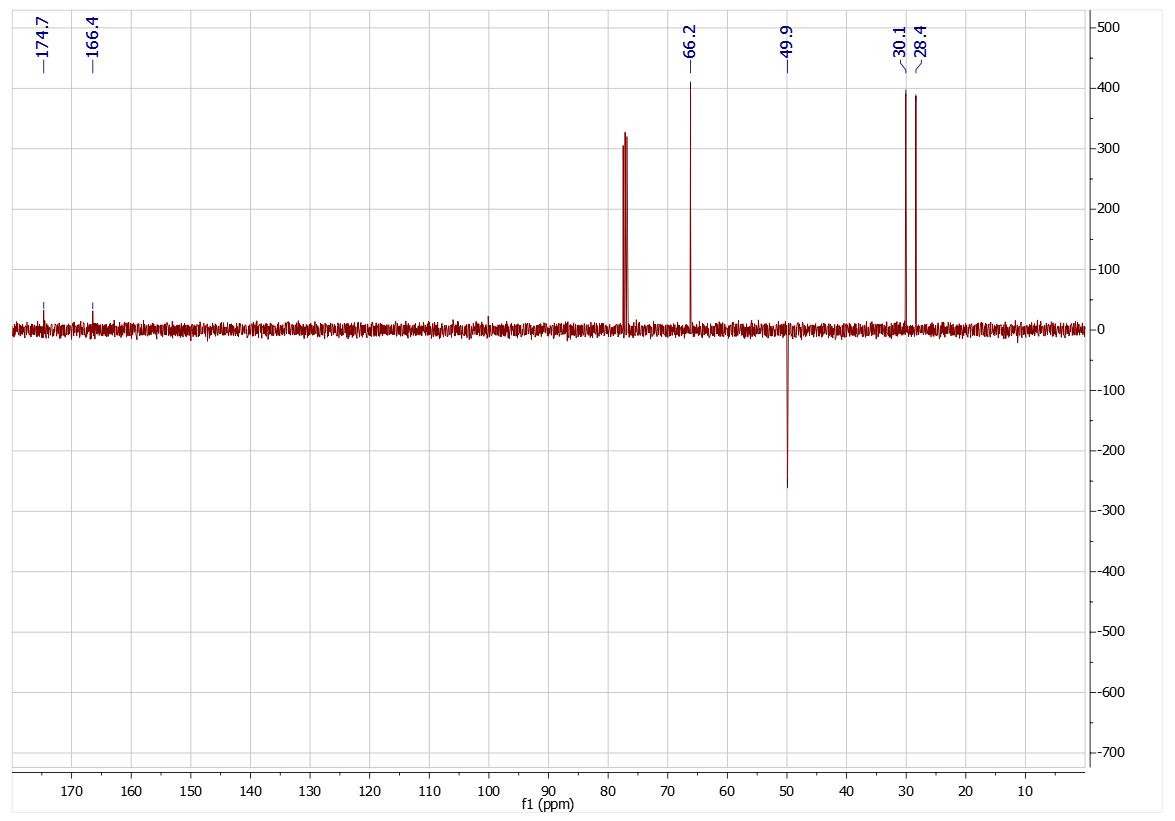

**Compound 7: 2-chloro-N-(2-oxotetrahydrofuran-3-yl)acetamide 7**

Chloroacetyl chloride (310 mg, 2.75 mmol, 0.22 mL), α-amino-γ-butyrolactone hydrobromide (500 mg, 2.75 mmol), triethylamine (556 mg, 5.49 mmol), chloroform (20 mL). Column chromatography (50% EtOAc in chloroform) gave **7** (334 mg, 1.88 mmol) in 68% yield as a white solid. **δ_H_** 7.05 (1H, br s, NH), 4.58 (1H, ddd, *J* 6.6, 8.6, 11.4 Hz, CH), 4.50 (1H, br t, *J* 9.0 Hz, CH), 4.31 (1H, ddd, *J* 5.9, 9.7, 10.8 Hz, CH), 4.10 (2H, s, CH_2_), 2.81-2.88 (1H, m, CH), 2.17-2.28 (1H, m, 1H); **δ_C_**174.6, 166.8, 66.1, 49.6, 42.3, 30.1; ***v*_max_** 3251, 3069, 1762, 1662, 1556, 1179, 1024; **MS(CI)** 178.0 (100%, [C_6_H_8_^35^ClNO_3_+H]^+^) 180.0 (30%, [C_6_H_8_^37^ClNO_3_+H]^+^), 200.0 (100%, [C_6_H_8_^35^ClNO_3_+Na]^+^), 202.0 (30%, [C_6_H_8_^37^ClNO_3_+Na]^+^); HRMS(ES) found 178.0264, C_6_H_9_^37^ClNO_3_^+^ ([M+H]^+^) requires 178.0265.


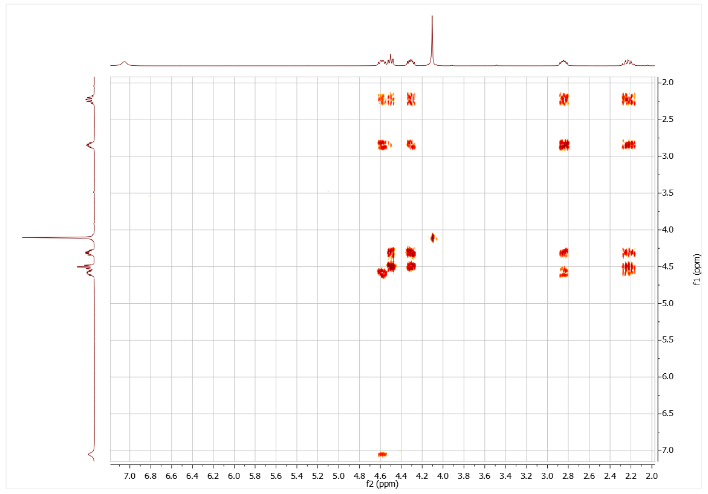

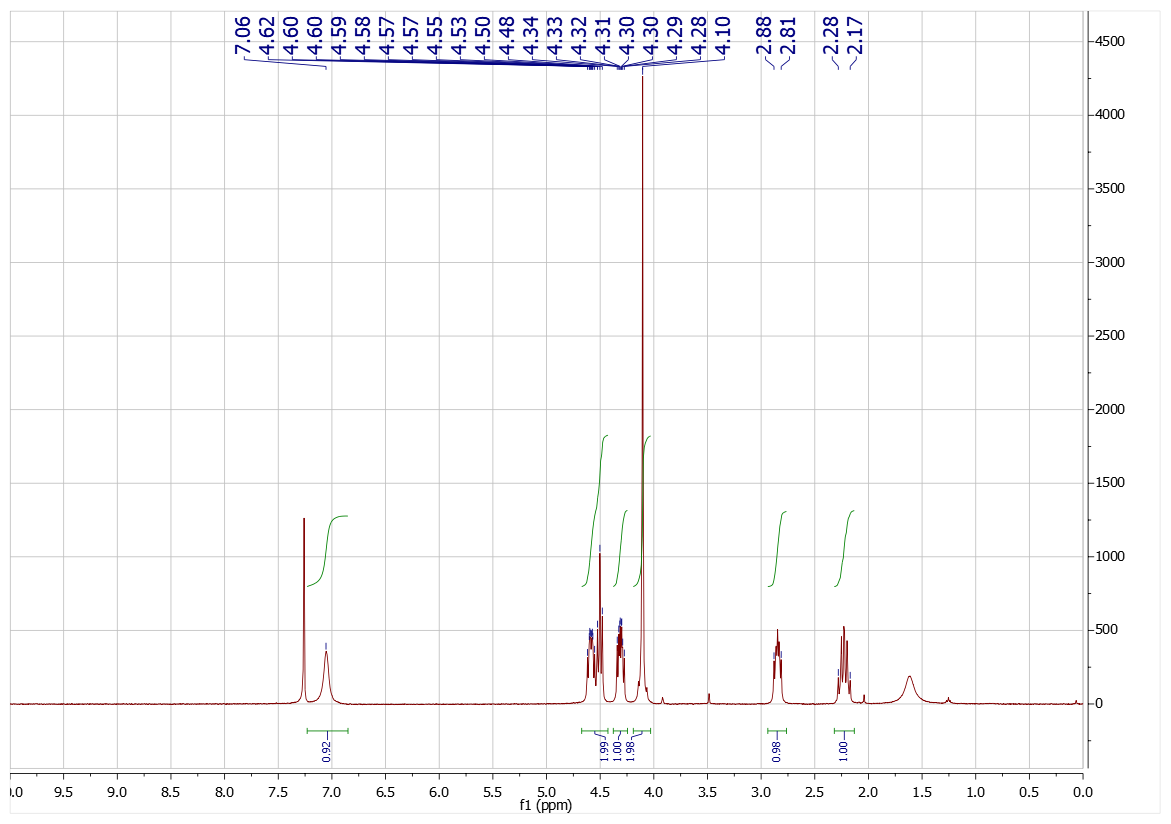

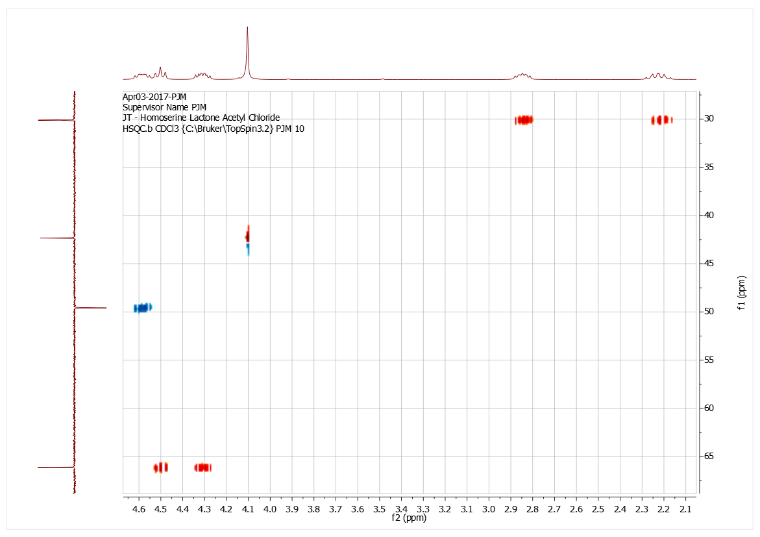

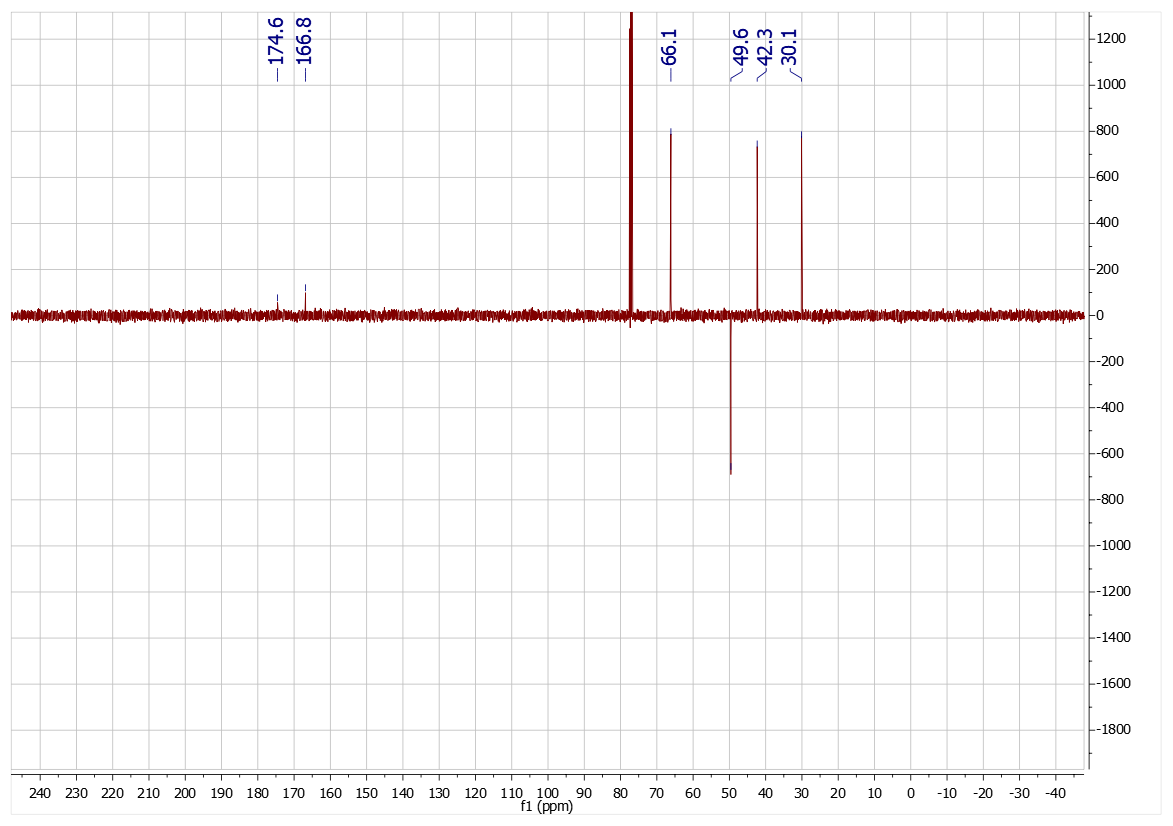

**Compound 8: 2-chloro-N-(2-oxotetrahydrothiophen-3-yl)acetamide 8**

Chloroacetyl chloride (753 mg, 6.51 mmol, 0.52 mL), homocysteine thiolactone hydrochloride (500 mg, 3.25 mmol), potassium carbonate (1.35 g, 9.76 mmol), water (20 mL), chloroform (20 mL). Column chromatography (50% EA in CF) gave **8** (382 mg, 1.97 mmol) in 61% yield as a white solid. **δ_H_** 6.95 (1H, br s, NH), 4.52 (1H, apparent pentet, 6.5 Hz, CH), 4.12 (1H, d, *J* 15.1 Hz, CH), 4.07 (1H, d, *J* 15.1 Hz, CH), 3.39 (1H, ddd, *J* 5.1, 11.6, 11.6 Hz, CH), 3.29 (1H, br dd, *J* 7.0, 11.2 Hz, 1H), 2.91-2.98 (1H, m, CH), 2.00 (1H, dddd, *J* 7.0, 12.5, 12.5, 12.5 Hz, 1H); **δ_C_** 204.5, 166.7, 59.7, 42.5, 31.7, 27.7; ***v*_max_** 3293, 2941, 1702, 1643, 1534, 1262; MS(CI) 194.0 (100%, [C_6_H_8_^35^ClNO_2_S+H]^+^) 196.0 (35%, [C_6_H_8_^37^ClNO_2_S +H]^+^), 216.0 (65%, [C_6_H_8_^35^ClNO_2_S +Na]^+^), 218.0 (23%, [C_6_H_8_^37^ClNO_2_S +Na]^+^); HRMS(ES) found 194.0037, C_6_H_9_^35^ClNO_2_S^+^ ([M+H]^+^) requires 194.0037.


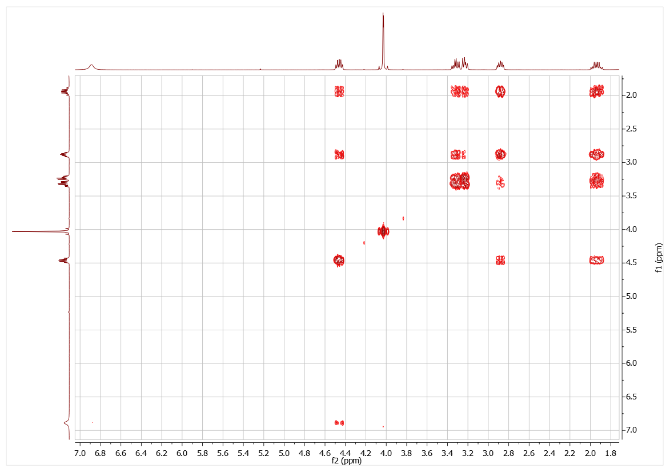

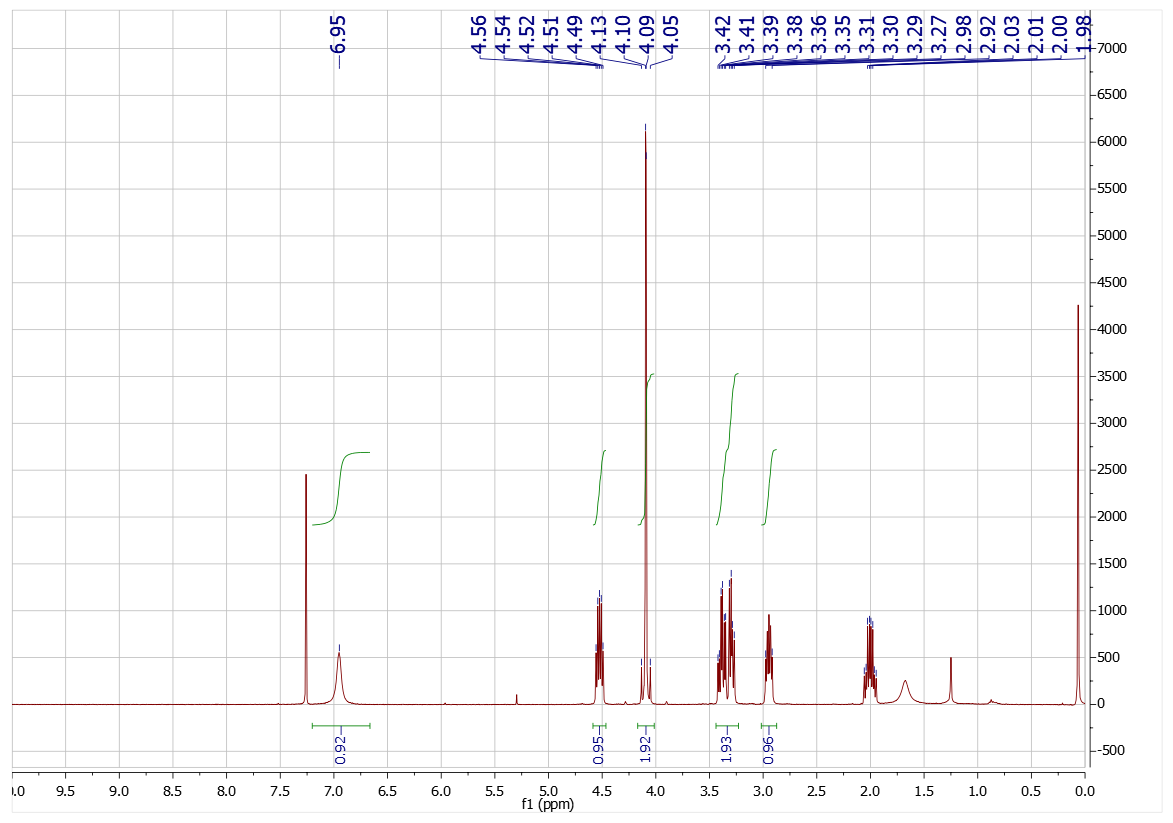

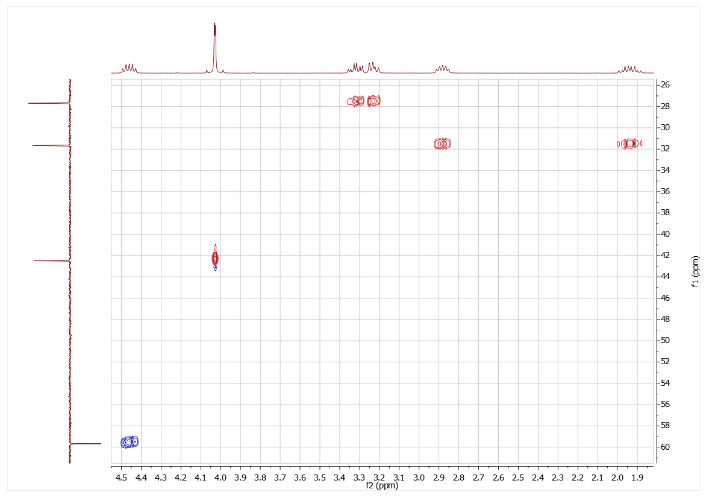

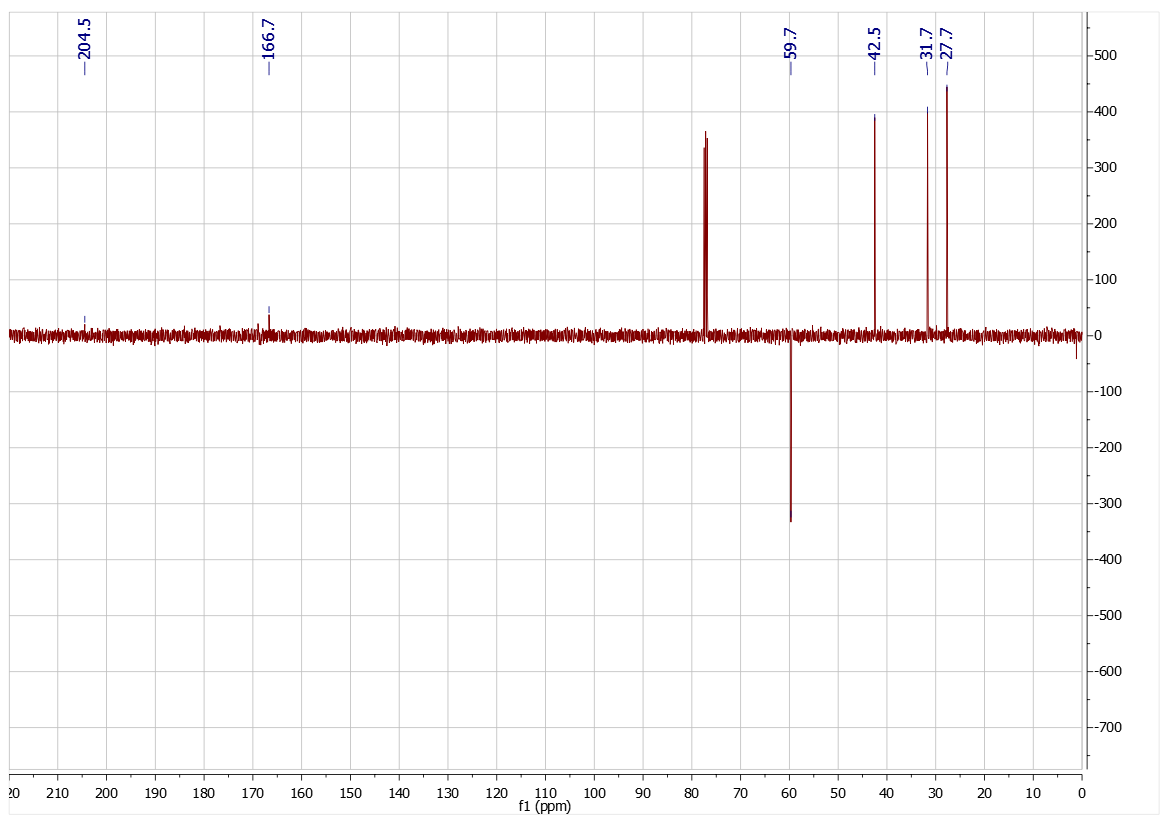

**Compound 9: 2-chloro-N-(2-oxotetrahydrofuran-3-yl)propanamide 9**

Chloropropianoyl chloride (349 mg, 2.75 mmol, 0.27 mL), α-amino-γ-butyrolactone hydrobromide(500 mg, 2.75 mmol) and triethylamine (556 mg, 5.49 mmol), chloroform (20 mL). Column chromatography (50% EtOAc in chloroform) gave **9** (341 mg, 1.78 mmol) in 65% yield as a white solid (1:1 mixture of diastereoisomers). **δ_H_** 6.99 (1H, br s, NH), 4.36-4.52 (3H, m, 3 x CH), 4.21-4.27 (1H, m, CH), 2.74-2.81 (1H, m, CH), 2.09-2.20 (1H, m, CH), 1.70/1.69 (3H, 2 x d, *J* 6.8/6.7 Hz, Me); **δ_C_** 174.7/174.7, 170.4/170.4, 66.1/66.1, 55.3/55.2, 49.6, 30.1/30.1, 22.5; ***v*_max_** 3285, 3085, 1775, 1660, 1551, 1167; MS(CI) 192.0 (100%, [C_7_H_10_^35^ClNO_3_+H]^+^) 194.0 (35%, [C_7_H_10_^37^ClNO_3_+H]^+^), 214.0 (85%, [C_7_H_10_^35^ClNO_3_+Na]^+^), 216.0 (25%, [C_7_H_10_^37^ClNO_3_+Na]^+^); HRMS(ES) found 192.0422, C_7_H_11_^79^ClNO_3_^+^ ([M+H]^+^) requires 192.0422.


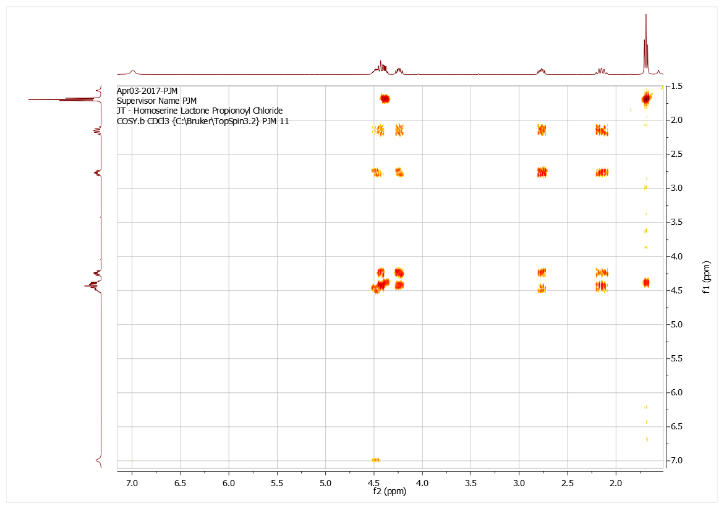

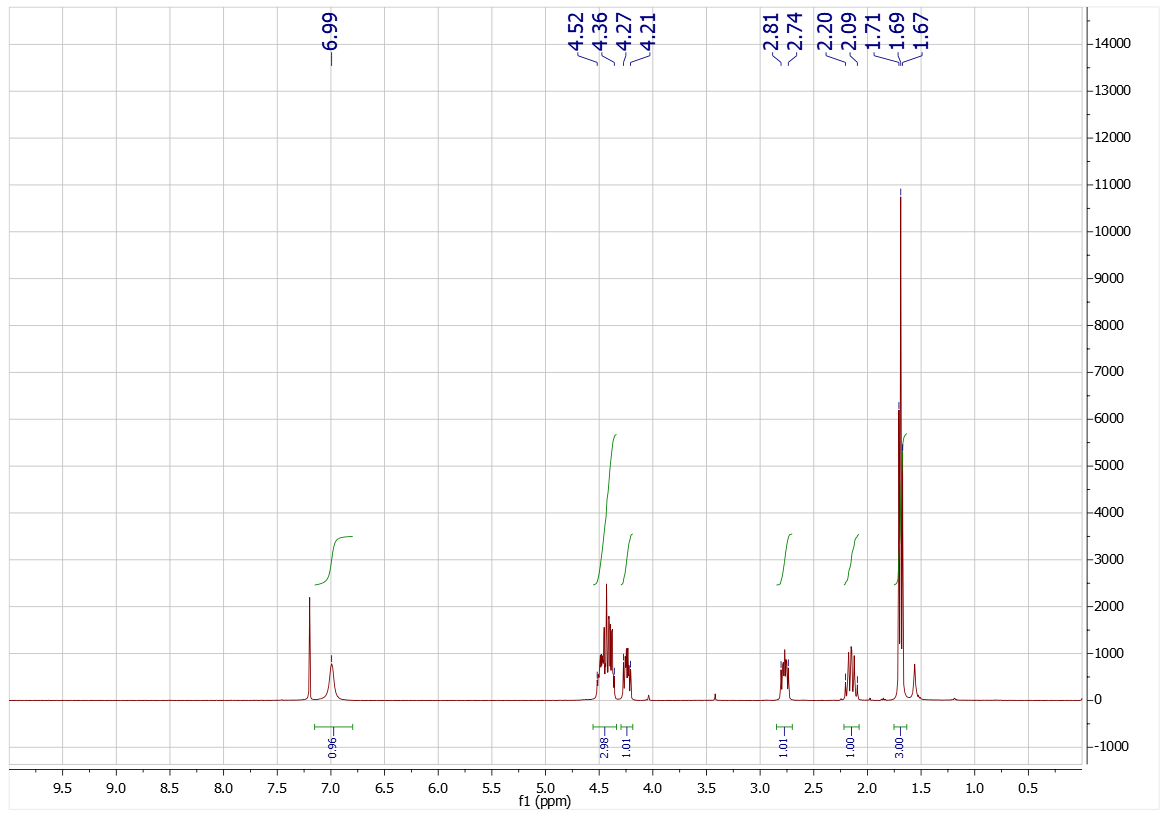

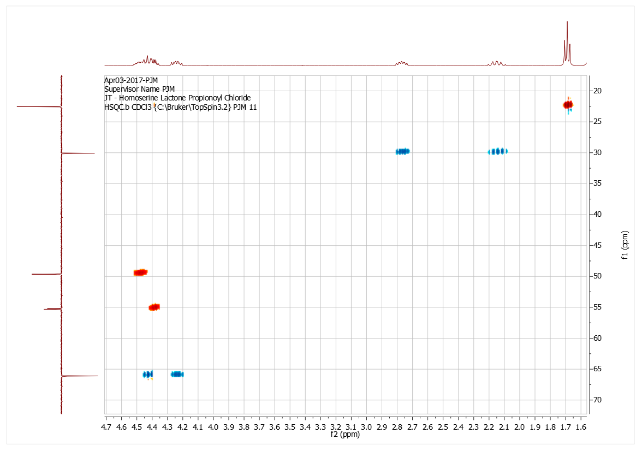

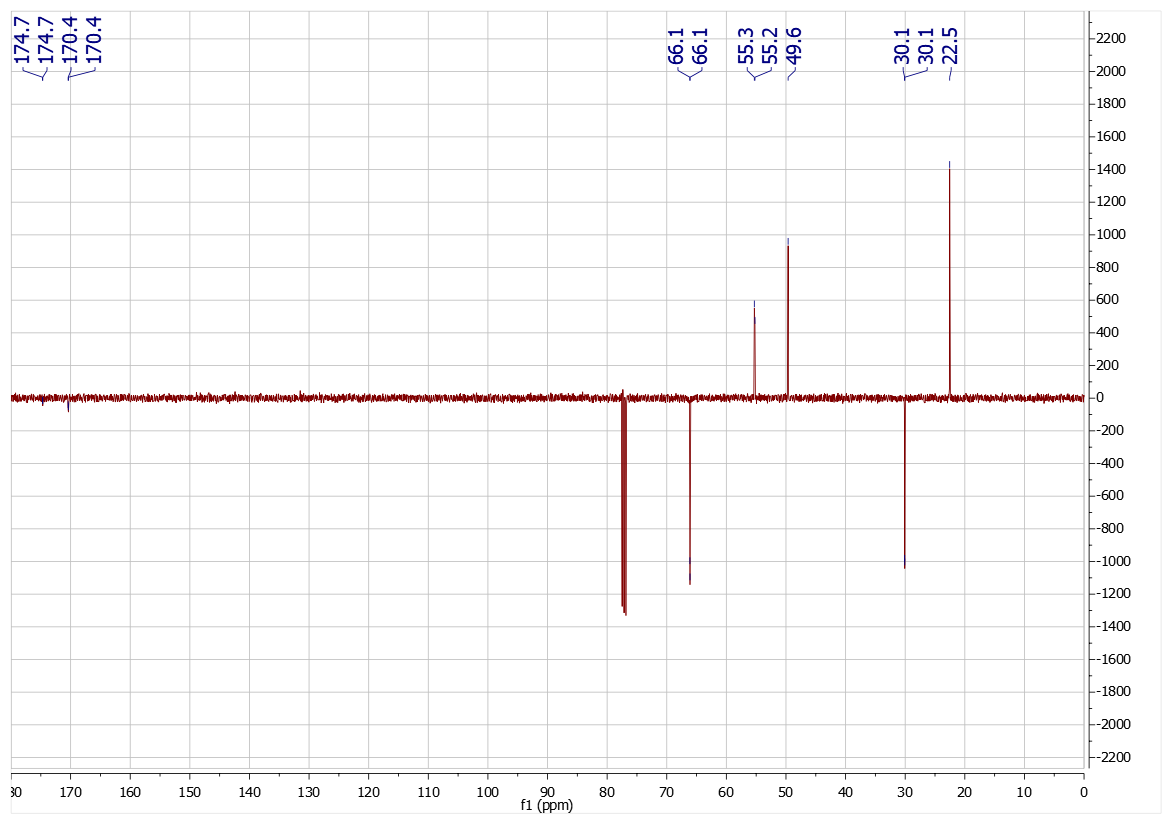

Supplement: S1 Protocol — (DOCX) [file pntd.0008630.s001.docx]
