# Supplementary figures and images for: Quorum sensing N-Acyl homoserine lactones are a new class of anti-schistosomal
Source: PLoS Negl Trop Dis. 2020 Oct 19;14(10):e0008630. doi: 10.1371/journal.pntd.0008630 (PMC7595621; doi:10.1371/journal.pntd.0008630)

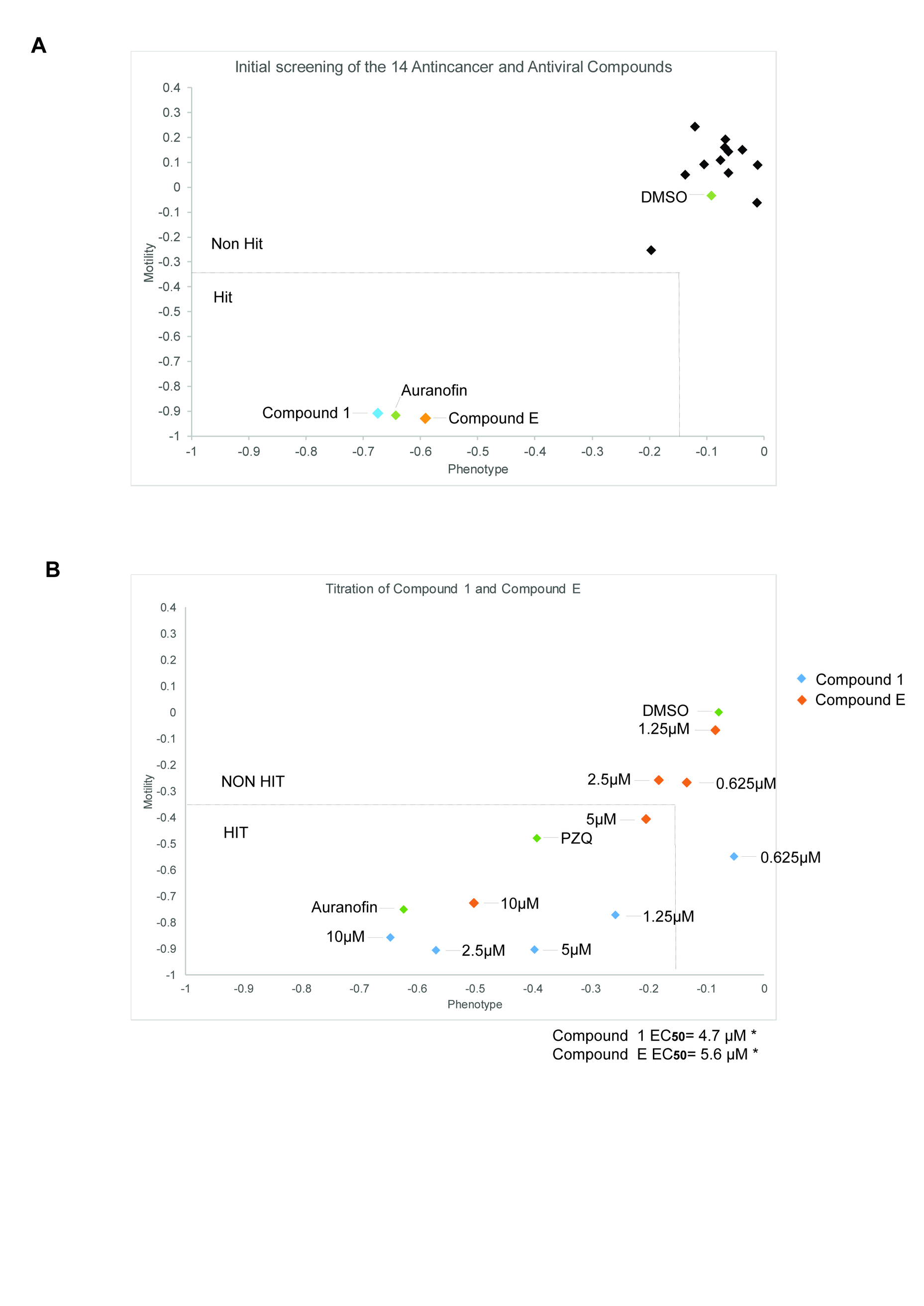

Supplement: S1 Fig — A total of 120 mechanically transformed schistosomula were co-cultured with each compound, titrated at doses between 10 and 0.625 μM. Test plates were incubated at 37°C for 72 hrs in an atmosphere containing 5% CO2. At 72hrs, schistosomula were scored using the Roboworm platform for both motility and phenotype as previously described [17, 25]. A) Of the 14 compounds evaluated, two fell within the hit region. Z’ scores for motility and phenotype were 0.40079 and 0.56846 respectively. B) Further titration of the two hit compounds resulted in good compound effect being observed for compound 1 down to a concentration of 1.25 μM. Z´ scores for motility and phenotype were 0.37864 and 0.47882 respectively. *Average EC50 value across motility and phenotype is presented for each compound. (TIF) [file pntd.0008630.s003.tif]
